# Structural Insights from Binding Poses of CCR2 and CCR5 with Clinically Important Antagonists: A Combined *In Silico* Study

**DOI:** 10.1371/journal.pone.0032864

**Published:** 2012-03-27

**Authors:** Gugan Kothandan, Changdev G. Gadhe, Seung Joo Cho

**Affiliations:** 1 Department of Bio-New Drug Development, College of Medicine, Chosun University, Gwangju, Korea; 2 Department of Cellular Molecular Medicine, Research Center for Resistant Cells, College of Medicine, Chosun University, Gwangju, Korea; Bioinformatics Institute, Singapore

## Abstract

Chemokine receptors are G protein-coupled receptors that contain seven transmembrane domains. In particular, CCR2 and CCR5 and their ligands have been implicated in the pathophysiology of a number of diseases, including rheumatoid arthritis and multiple sclerosis. Based on their roles in disease, they have been attractive targets for the pharmaceutical industry, and furthermore, targeting both CCR2 and CCR5 can be a useful strategy. Owing to the importance of these receptors, information regarding the binding site is of prime importance. Structural studies have been hampered due to the lack of X-ray crystal structures, and templates with close homologs for comparative modeling. Most of the previous models were based on the bovine rhodopsin and β2-adrenergic receptor. In this study, based on a closer homolog with higher resolution (CXCR4, PDB code: 3ODU 2.5 Å), we constructed three-dimensional models. The main aim of this study was to provide relevant information on binding sites of these receptors. Molecular dynamics simulation was done to refine the homology models and PROCHECK results indicated that the models were reasonable. Here, binding poses were checked with some established inhibitors of high pharmaceutical importance against the modeled receptors. Analysis of interaction modes gave an integrated interpretation with detailed structural information. The binding poses confirmed that the acidic residues Glu291 (CCR2) and Glu283 (CCR5) are important, and we also found some additional residues. Comparisons of binding sites of CCR2/CCR5 were done sequentially and also by docking a potent dual antagonist. Our results can be a starting point for further structure-based drug design.

## Introduction

Chemokines are small (8–10 kDa) water-soluble proteins consisting of 340–380 amino acid residues, which play key roles in immuno-modulation and host defense. They selectively recruit monocytes, neutrophils, and lymphocytes to sites of vascular injury and inflammation [1]–[3]. Different chemokines produce various leukocyte responses depending on the complementary nature of their chemokine receptors [4], [5]. The basic feature of inflammation is the tissue recruitment of leukocytes, which is mediated mainly by chemokines (chemotactic cytokines) via their receptors. The chemokine super family can be categorized into four groups (CC, CXC, CX3C, and C), according to the number and spacing of conserved cysteines in the amino acid sequence [6]–[9]. Apart from their well-recognized role in leukocyte recruitment, some chemokines and chemokine receptors play crucial roles in other cellular functions such as activation, proliferation, and differentiation [6]–[9]. Specific family members are also involved in viral entry and angiogenesis [9]. It was also reported that, a subset of chemokine receptors plays a non-redundant role in infectious diseases, as demonstrated by resistance to human immunodeficiency virus/acquired immunodeficiency syndrome (HIV/AIDS) in people homozygous for CCR5 Δ 32 (a loss of function mutation) [10]–[14].

Because of their diverse range of important functions, chemokines have been targeted as potential points of pharmaceutical intervention for diseases as diverse as asthma, rheumatoid arthritis, multiple sclerosis, solid organ transplantation, atherosclerosis, cancer, and HIV infection [9]. Since these chemokine receptors are G protein-coupled receptors and targeted for diverse diseases, many pharmaceutical and biotechnology companies have devoted enormous time, effort, and expense in developing potent small-molecule chemokine antagonists [15], [16]. Accordingly, use of two such antagonists, Maraviroc (a CCR5 antagonist) for the treatment of HIV/AIDS [17] and Plerixafor (a CXCR4 antagonist) used in combination with granulocyte-colony stimulating factor (G-CSF) to mobilize hematopoietic stem cells to the peripheral blood for collection and subsequent autologous transplantation in patients with non-Hodgkin's lymphoma and multiple myeloma have been approved by the United States Food and Drug Administration (FDA) [18].

But, for chronic inflammatory diseases, clinical trials with antagonists of a single chemokine receptor (e.g., CCR1, CCR2, or CCR5) have not proved successful [15], [16], which has been a major setback. Considering the difficulty of pathogenesis of these diseases and the potential for functional redundancy of chemokine receptors, targeting a single receptor may not be adequate for efficacy for these chronic conditions. CCR2 and CCR5 are two CC chemokine receptors that are important players in the trafficking of monocytes/macrophages and in the functions of other cell types relevant to disease pathogenesis [19], [20]. So, structural information of CCR2 and CCR5 can be useful and essential for providing insights about targeting these receptors. Two recent studies have reported the use of dual antagonists targeting both CCR2 and CCR5 [21], [22].

Computational modeling has become an essential tool in guiding and enabling rational decisions with respect to hypothesis-driven biological research. In the absence of an experimentally determined structure, homology modeling can provide a rational alternative to a reasonable 3D structure. Knowledge of the 3D structure of these receptors is important for understanding the underlying molecular mechanisms of diseases caused by mutations. Also, 3D structures will provide an opportunity for structure-based drug design of small molecules acting as potent antagonist and, provides the opportunity for site-directed mutagenesis studies.

The aim of this study was to provide adequate information regarding the binding site of CCR2 and CCR5 receptors. We used various computational techniques such as homology modeling, docking, and molecular dynamic simulations (MDS). Homology modeling of CCR2 and CCR5 was done using the crystal structure of CCXR4 as the template [23]. These homology models were further refined using MDS, and docking was done for the potent antagonists of CCR2 and CCR5 against the modeled receptor structure. Also, active sites of CCR2 and CCR5 were compared. A potent dual antagonist was docked into the active site (CCR2/CCR5) and results were analyzed.

## Materials and Methods

### Sequence analysis of CCR2 and CCR5

The human sequences of CCR2 and CCR5 were retrieved from the Uniprot KB/TrEMBL database (accession numbers P41597 and P51681). In order to identify an adequate template for modeling of CCR2 and CCR5 chemokine receptors, the Basic local alignment search tool for protein (BLAST) algorithm [24], [25] was carried out against the protein data bank [26]. After the search, the alignment between the template and the target sequences (CCR2 and CCR5) was performed using ClustalW 2.0 [27] with default parameters.

### Comparative modeling of CCR2 and CCR5

A number of homologous structures were identified as templates in the protein data bank. Among the available templates from search, CXCR4 (protein data base code: 3ODU; resolution-2.5 Å) [23] was found as top template, and subsequently comparative modeling was done. With the given identified hit as template structure, sequence alignments for query sequences (P41597 and P51681) were carried out. The structures of both CCR2 and CCR5 were generated using the Modeller9v4 program [28]–[30]. Modeller9v4 calculates a model composed of non-hydrogen atoms, based on the alignment of the sequence to be modeled with known related structures. A 3D model was obtained by optimization of a molecular probability density function (PDF) using a variable target function procedure in Cartesian space that employs methods of conjugate gradients and molecular dynamics with simulated annealing. One hundred 3D models were generated for both CCR2 and CCR5, and the models with lower molecular probability density function (Molpdf) score and lower root mean square deviation (RMSD) value were selected for further computational study. The selected CCR2 and CCR5 models were refined by molecular dynamic simulations and were further validated using PROCHECK [31], ERRAT [32] and ProSA (https://prosa.services.came.sbg.ac.at/prosa.php) analyses.

### Molecular dynamics simulation (MDS)

MDS were performed to produce good starting structures for docking study. At this stage, the minimum energy conformers of CCR2 and CCR5 obtained from comparative modeling were used as the starting structures for MD simulation. Protein and water molecules were used as the components for the simulation.

To remove bad contacts of the modeled receptors and to achieve good starting structures, the models (CCR2 and CCR5) were refined using MDS of 5000 ps with the GROMACS package using the GROMOS96 force field [33]. The initial structures (CCR2 and CCR5) were placed in a 0.9 nm cubic box. The extracellular regions of the receptor are hydrophilic in nature, whereas the transmembrane domains are hydrophobic in nature. As the TM's are hydrophobic, care was taken that no water molecules are present in those regions. The SPC water model [34], [35] was used to create the aqueous environment for both models. Periodic boundary conditions were applied and the systems were further neutralized by adding appropriate counter ions (Na+ and Cl−). The system was then subjected to 500 steps of energy minimization using a steepest descent algorithm [36] to reduce the effect of unfavorable interactions produced by generation of solvents and ions.

The models (CCR2 and CCR5) were further subjected to full MDS for a period of 5000 ps without restraints. During this phase, (NVT) and (NPT) ensembles were used. The Berendsen coupling scheme was used with both ensembles. All bond lengths were constrained using the LINCS algorithm [37]. The SETTLE algorithm was used to constrain the geometry of water molecules [38].

### Binding site construction and docking analysis

**Figure 1:**
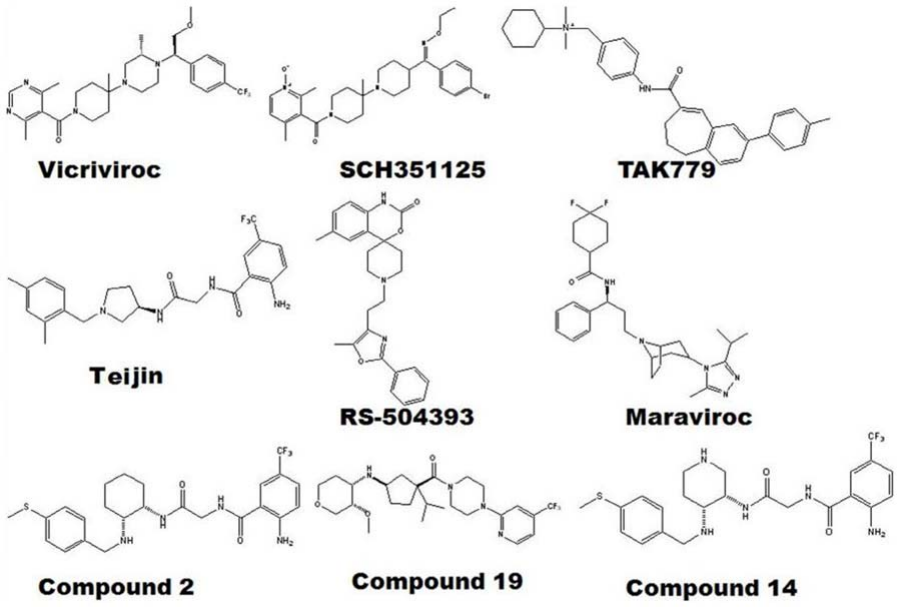
Chemical structures of studied compounds using molecular docking. CCR2 (Compound 2, 14, RS-504393 and Teijin), CCR5 (Maraviroc, SCH351125, TAK779 and Vicriviroc) and dual inhibitors (Compound 19).

The Autodock 4.0 program was used for docking calculations. Autodock uses the Lamarckian genetic algorithm (LGA) and is regarded as the best method in terms of its ability to deduce the lowest energy structure and the accuracy of its structure predictions [39]. Hydrogen atoms and the active torsions of ligands were assigned using Autodock tools (ADT). The binding site for the receptor structures (CCR2 and CCR5) was created according to previously published results. With this prior knowledge, the binding site was created within 5 Å. An autogrid was further employed to generate grid maps around the active site with 60×60×60 points and grid spacing set to 0.375 Å. Docking parameters modified from the defaults were the number of individuals in the population (set to 150), maximum number of energy evaluations (set to 2,500,000), maximum number of generations (set to 27,000), and number of GA runs (set to 20). The final conformations were clustered and ranked according to the Autodock scoring function as well as with the knowledge of crucial residues determined by mutational studies and experimental analysis. In this study, the binding mode of some of the potent inhibitors reported for CCR2 and CCR5 were determined and analyzed (Figure 1).

## Results

### Sequence analysis of CCR2 and CCR5

**Figure 2:**
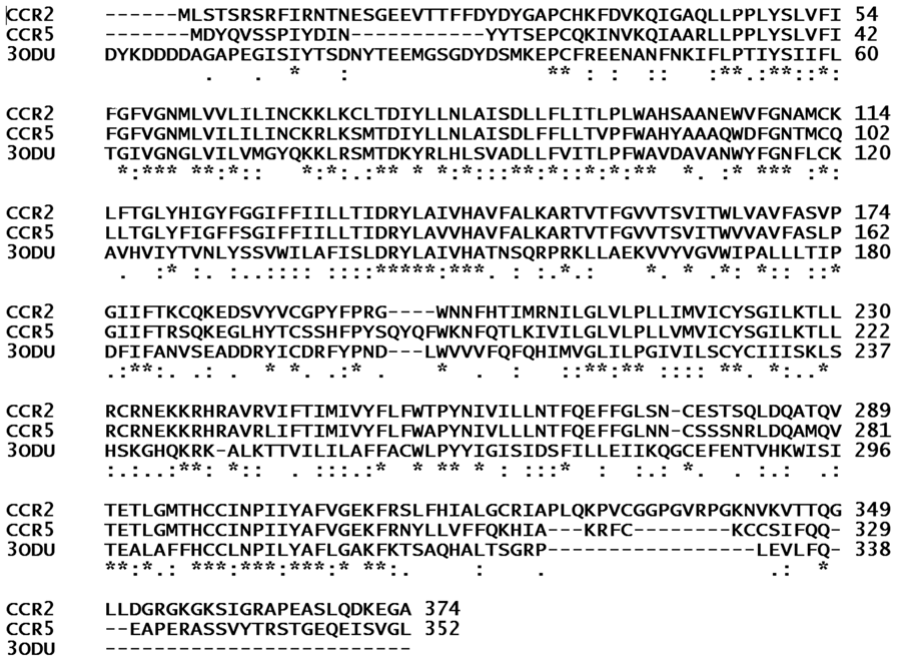
Sequence alignment of CCR2 (UniProtKB: P41597) and CCR5 (UniProtKB: P51681) with the CXCR4 (PDB ID; 3ODU) as template. Star indicates identical amino acids; colon indicates similar amino acids and single dot designate almost similar amino acid.

A BLAST search revealed 35% sequence identities between template (3ODU) and query sequences (CCR2 and CCR5), and a 60% identity between the active sites of template and query. The sequences showed a high level of homology between the target and template sequences and were better than that of the traditional bovine rhodopsin and the more recent β2-adrenergic receptor templates. The obvious reason for this is the template sequences are from the close homologue (CXCR4). The more significant step in the modeling procedure is to obtain an acceptable alignment of the target with the template sequences. This was performed and the alignment obtained using ClustalW 2.0 is shown in Figure 2. The expectation value (E-value) represents a number of different alignments with scores equivalent to or better than the scores that are expected to occur in a random database search. Generally, a lower E-value indicates that alignment is real and does not occur by chance. The E-value for CCR2 and CCR5 was 2e-33 and 1e-33, respectively.

### Homology modeling

The A-chain of CXCR4 was used to develop the 3D models and a modeler program was used to derive 3D-models of CCR2 and CCR5. In the models, the seven-transmembrane (TM) helixes are correctly transformed according to that of the template (3ODU) structure. One hundred models were developed for both CCR2 and CCR5. Finally, a model (CCR2 and CCR5) with a lower MolPdf value and the one that displayed a lesser RMSD was selected for further computational analysis. More than 90% of the members of the GPCR super family have conserved disulfide bridges. In CCR2, disulfide bridges were created between Cys32–Cys277 and Cys113–Cys190. In CCR5, the disulfide bridges were maintained between Cys20–Cys269 and Cys101–Cys178. The selected models were further validated stereo-chemically using additional parameters such as PROCHECK [31]. The Ramachandran plot for model before refinement by MDS is shown in the materials S1 and S2.

### Molecular dynamics simulation

**Figure 3:**
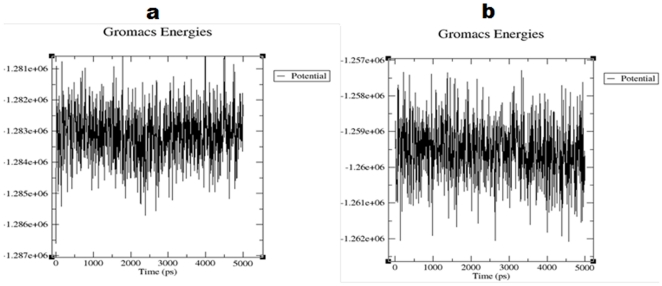
Potential energy plot of MD simulation. (a) CCR2 and (b) CCR5 plot shows the variation in potential energy throughout the system for a period of 5 ns. Time is on the X-axis and the potential energy is on the Y-axis.

The models (CCR2 and CCR5) selected from modeler was further refined by MDS, to improve and access the stability of the model. We also implemented MDS to find the energetically favorable structure for further docking analysis. Our analysis based on the trajectory revealed that the potential energy of the model (CCR2) decreased from −1.281e+06 KJ/mol to −1.286e+06 KJ/mol. However, in the case of CCR5, a decrease in the potential energy was more and it varied from −1.257e+06 KJ/mol to −1.261e+06 KJ/mol. Most of the structures are around the area of −1.283e+06 KJ/mol and −1.259e+06 KJ/mol for CCR2 and CCR5, respectively. These data indicate the energetic stability of CCR2 and CCR5. The potential energy plot for CCR2 and CCR5 is shown in Figure 3.

**Figure 4:**
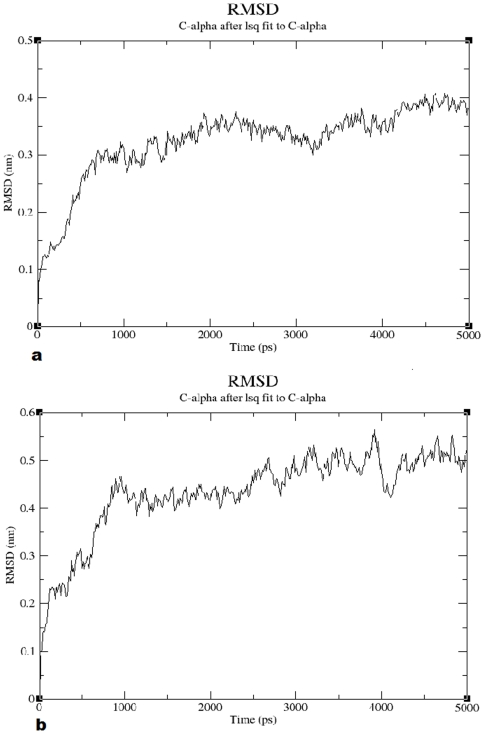
Graphical representation of root mean square deviation (RMSD) plot. RMSD for (a) CCR2 and (b) CCR5 Cα from the initial structures throughout the simulation of 5 ns as function of time.

The models were also evaluated on the basis of structural stability using the RMSD calculated by variation in structure with respect to time. The first 1000 ps were considered as the period of equilibration. For CCR2, there was a gradual rise until 0.35 nm followed by a plateau (Figure 4a). For CCR5, a gradual rise was observed until 0.45 nm and a plateau was observed throughout the rest of the period (Figure 4b). These results also indicate the structural stability of the models.

**Figure 5:**
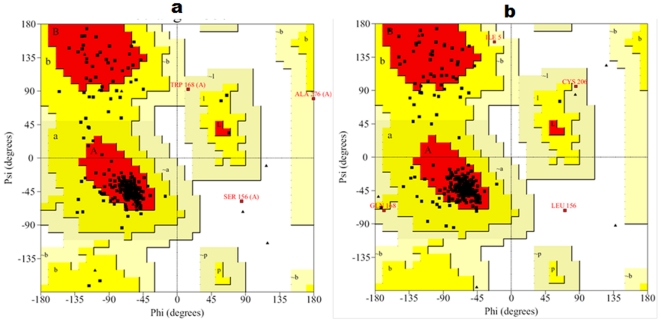
Ramachandran plot for the models after MD simulations. (a) CCR2 and (b) CCR5 models are shown. The different color coding indicates most favored (red), generously allowed (dark yellow), additionally allowed (light yellow), and disallowed (white) regions.

One of the lowest potential energy conformations of CCR2 and CCR5 was selected and refined by simple minimization. The selected models were further validated stereo-chemically using PROCHECK [31] and ERRAT [32] plots. The statistical parameters obtained for both the CCR2 and CCR5 models are summarized in Table 1. Ramachandran plot for the CCR2 model showed that most of the residues are in mostly favored and additionally allowed region. Similarly, in case of CCR5, the residues are in mostly favored additionally allowed and generously allowed regions. MDS shifted only one residue of CCR2 (Ser156) and CCR5 (Leu159) into the disallowed region. Analyses of both the structures for particular residues indicated that none of the residues are part of the active site. The Ramachandran plot for the CCR2 and CCR5 models is shown in Figure 5.

**Table 1:** Validation results of CCR2/CCR5 homology model before and after MDS.

	PROCHECK				
Model					ERRAT %
	Core %	Additionally allowed %	Generously allowed %	Disallowed %	
CCR2 (Before MD)	92.9	7.1	0	0	87.04
CCR2 (After MD)	84.1	14.7	0.8	0.4	89.92
CCR5 (Before MD)	92.8	6.8	0.4	0	88.41
CCR5 (After MD)	84.8	13.7	1.1	0.4	90.78

ERRAT plot analysis indicated that the overall quality factor for non-bonded atomic interactions between atom types. Models with higher ERRAT score indicate the model with higher quality. Presently, the ERRAT score was found to be 89.92 for CCR2 and 90.78 for CCR5 and it is better than those models which were obtained before MDS which indicating the quality of the generated models. In addition to this, we also validated our models using Prosa which evaluates the energy of the structure using distance pair potential. Residues with negative Prosa score confirm the reliability of the model. The Prosa energy score for the template was found to be -2.34 and for the models it was found to be better (CCR2: Before MD - 2.54, After MD - 2.80; CCR5: Before MD - 2.80, After MD - 2.93). The Prosa energy plot is shown in materials S3.

Overall, our results indicate the selected models are satisfactory. The quality of the model was evident by ERRAT score as well as the Prosa energy scores. On the other hand, results PROCHECK showed slightly worsened values of selected models. However, from the results we can conclude that almost all the residues are in most favored and additionally allowed regions except a single residue in both CCR2 and CCR5. Both the residues do not have prime importance and most importantly, the active site residues are well within the limits of Ramachandran plot. Moreover, a slight drift in the RMSD of the protein models after MDS is quite common and it is evident in the literature. After a period of equilibration, the structures were found to be stable throughout the simulation (Figure 4a and Figure 4b). These refined models of CCR2 and CCR5 were further used for docking analyses and are shown in materials **S4** and **S5**, respectively.

### Prediction of interaction between potent CCR2 antagonists and CCR2 receptor

#### Binding site of CCR2

**Figure 6:**
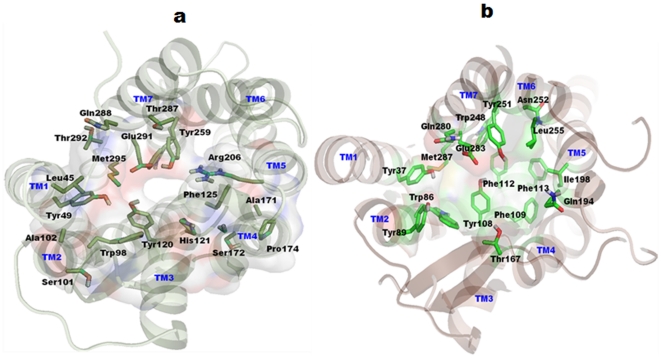
Top views of putative binding pockets after MD simulation for docking analyses. (a) CCR2 transmembrane (TM) helices are shown in light green, whereas, constructed binding pocket residues were shown in smudge green sticks. All the TM regions are labeled by blue color on the top of helices. The binding pocket is also represented as transparent molecular surfaces. (b) CCR5 TM helices are shown in light brown color, whereas constructed binding pocket residues were shown in green sticks. Figure generated using Pymol program (http://www.pymol.org).

Receptor homology modeling suggests that the antagonists bind in an extended pocket bounded by TM2, TM3, TM5, TM6, and TM7. It has been proposed from mutagenesis studies that Glu291 from TM7 is an important residue in the binding pocket [4], [40]. With the knowledge of these previously published results, the binding pocket was determined. The binding pocket was composed mainly of residues Phe35, Val37, Leu45, Tyr49, Trp98, Ser101, Ala102, Tyr120, His121, Tyr124, Phe125, Ala171, Ser172, Pro174, Gly175, Val189, Phe194, Arg206, Asn207, Trp256, Tyr259, Gln288, Glu291, Thr292, and Met295, similar to previous studies [4], [40]. The residues that guided docking are shown in Figure 6a. As the proposed binding pocket was similar, some of the potent CCR2 antagonists were docked into the binding site.

#### Docking studies of CCR2 antagonists

A wide variety of structurally diverse small molecule antagonists have been reported in the literature. However, only few of them are reported using a combined in silico analysis and mutagenesis studies to propose the binding site of CCR2 [4], [40]. In this study, we used potent antagonists such as (R)-3-aminopyrrolidine (Teijin lead), cyclohexyl and pyridyl derivatives, and RS-504393. Binding energies of all docked CCR2 inhibitors are given in materials S6.

#### Docking studies of (R)-3-aminopyrrolidine (Teijin lead)

**Figure 7:**
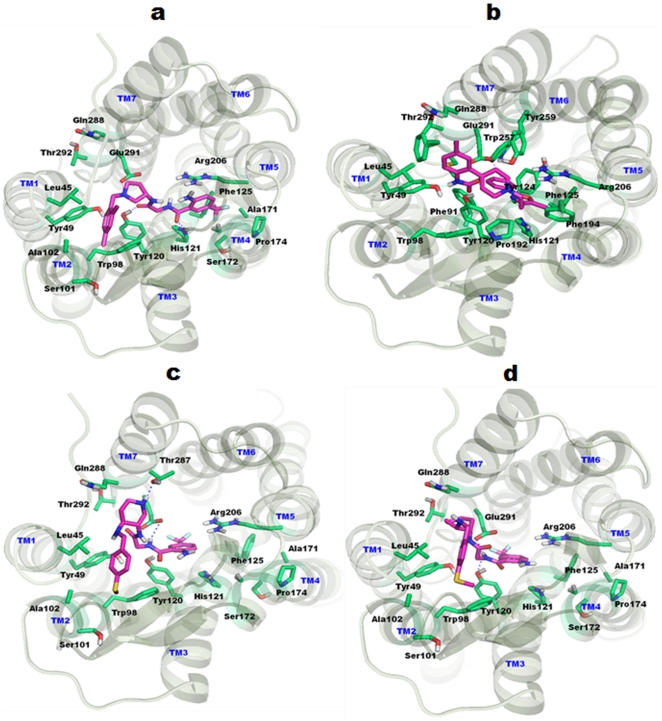
Binding modes of CCR2 inhibitors. TM helices are shown in pale green color, whereas constructed binding pocket residues were shown in cyan sticks. All the TM's are labeled by blue color on the top of helices. Docked ligands were shown in magenta color. (a) Docking model of Teijin shows key salt bridge interaction between pyrrolidine nitrogen and Glu291 by magenta dotted lines. Hydrogen bonding interactions are also observed with Tyr120 and His121. (b) RS-50323 shows salt bridge interaction between the linker nitrogen of the ligand and Glu291 which is indicated by magenta dotted lines. (c) Pyridyl derivative show crucial interaction between the hydrogen atom of the nitrogen and Glu291 which is indicated by magenta dotted lines. Hydrogen bonding interaction is also observed with Thr287. (d) Docking model of cyclohexyl derivatives identified crucial interaction between the hydrogen atom of nitrogen and Glu291 (magenta dotted lines). In addition, the same atom also hydrogen bonded with Tyr120.

A series of CCR2 antagonists have been reported [41]. The reported compounds were derivatives of the Teijin lead. The highly active compound of the (R)-3-aminopyrrolidine (IC_50_ = 3.2 nM) series was docked into the proposed binding site. Different conformations were generated and the conformations with the top cluster were selected. The ligand established crucial interactions with important residues in the binding site. The basic nitrogen in the pyrrolidine ring formed an electrostatic interaction (i.e., salt bridge) with crucial and conserved Glu291. The distance between the glutamic acid residue and the basic nitrogen was 3.95 Å. The ligand also formed hydrogen bond interactions with Tyr120 and His121. In addition to some of the hydrogen bonds, hydrophobic interactions were also observed between the ligand and the receptor. The 2,4-di-phenyl ring lay in the pocket lined by residues Tyr49, Trp98, and Ser101. Similarly, the trifluoro methyl group lay inside the cavity occupied by residues such as Phe125, Pro174, and Arg206. The binding mode of the ligand and its interaction with the receptor structure is shown in Figure 7a.

#### Docking studies of RS-504393

RS-504393 has been identified as a potent CCR2 antagonist [4]. The authors reported a group of spiropiperidine derivatives as potent antagonists against CCR2. Among them, RS-504393 was proposed as the most active one; we used this derivative (IC_50_ = 89 nM) for *in silico* docking simulations. The authors also found that the basic nitrogen present in the spiropiperidine compounds may be the interaction partner for Glu291. With this knowledge, the docking modes were analyzed and the binding pose was selected. The basic nitrogen in the spiropiperidine ring formed a salt bridge contact with Glu291 at a distance of 5.3 Å. Hydrogen bonding interactions were also observed between the ligand and the receptor. The other nitrogen of this spiropiperidine ring hydrogen bonded with Tyr49. The oxygen atom present in this ring also forms a hydrogen bond with Tyr120. Moreover additional hydrophobic interactions were observed between the ligand and the active site residues. The binding pose of the ligand and its interaction with the receptor structure is shown in Figure 7b.

#### Docking studies of cyclohexyl and pyridyl derivatives

Cherny et al. proposed a series of cyclohexyl and pyridyl derivatives as CCR2 antagonists [42]. They compared cyclohexane and piperidine derivatives and concluded that addition of the piperidine nitrogen alone can significantly enhance CCR2 affinity. Presently, we used one of the pyridyl (compound 14) derivatives (IC_50_ = 6.3 nM) and a cyclohexyl (compound 2) derivative (IC_50_ = 1180 nM) for docking analyses. Two Hydrogen bond interactions were observed between the receptor and the pyridyl derivative. The amide nitrogen of the pyridyl derivative, which is positioned close to the phenyl ring, interacts with the crucial Glu291. In addition, the nitrogen present in the piperidine ring interacts with Thr287 through hydrogen bond. The S-methyl group lies inside the pocket lined by residues Trp98, Ala101, Ser102, Val189, and Cys190. In case of the cyclohexyl derivative, the amide nitrogen interacts with Tyr120 and Glu291. Additional hydrophobic interactions were also observed between ligand and the receptor, similar to that of the pyridyl derivative. The binding pose of ligand and its interaction with the receptor structure is shown in Figure 7c and 7d.

### Prediction of interaction between potent CCR5 antagonists and CCR5 receptor

#### Binding site of CCR5

The binding pocket for CCR5 inhibitors was determined based on the previously published mutagenesis studies [43]–[47]. The binding pocket is located at extracellular region and is partly covered by the extracellular loop 2 (ECL2). It mainly composed of conserved residues Tyr37 (TM1), Trp86 (TM2), Tyr108 (TM3), Phe109 (TM3), Phe112 (TM3), Gln194 (TM5), Ile198 (TM5), Trp248 (TM6), Tyr251 (TM6), Gln280 (TM7), Glu283 (TM7), and Met287 (TM7). The binding pocket comprising residues along with TM regions are shown in Figure 6b. A literature review has suggested that the crucial acidic residue (Glu283) in TM7 of the binding pocket could establish ionic interactions with tertiary/quaternary nitrogen of inhibitors (Maraviroc, SCH-C, TAK779, and Vicriviroc).

#### Docking studies of CCR5 antagonists

A wide variety of potent and highly active CCR5 antagonists were used in docking studies. Potent CCR5 antagonists used in docking simulation included Maraviroc, SCH-C, TAK779, and Vicriviroc. The mutational data for all these compounds were previously reported and were collectively used to determine the binding pocket of CCR5. Binding energies of all docked CCR5 inhibitors are given in materials **S6**.

#### Docking study with Maraviroc

**Figure 8:**
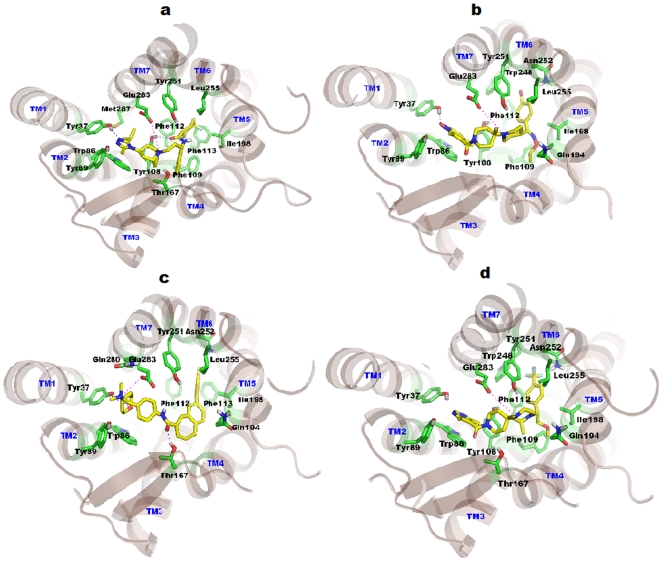
Binding modes of CCR5 inhibitors. TM helices are shown in light brown color, whereas constructed binding pocket residues were shown in green sticks. All the TM's are labeled by blue color on the top of helices. Docked ligands were shown in yellow color. (a) Docked pose of Maraviroc in CCR5, the key salt bridge interaction with Glu283 is shown by magenta dotted line. Hydrogen bonds with Try37 and Tyr108 were shown in blue dotted lines. (b) Docking model of SCH-C show a key salt bridge interaction with Glu283 and represented by magenta dotted line. Hydrogen bond with Try37 is shown as blue dotted lines. Pyridine-N-Oxide ring of ligand interacts through strong aromatic π-stacking interaction with the Trp86 of CCR5. (c) TAK779 in CCR5 shows salt bridge interaction with Glu283 which is designated by magenta dotted line. Hydrogen bonds with Try37 and Thr167 are shown in blue dotted lines. Phenyl group of TAK779 docked deeply inside the cavity formed by Ile198, Tyr251, Asn252 and Leu255. (d) Docking model of Vicriviroc shows salt bridge interaction with Glu283 which is indicated by magenta dotted line. Pyrimidine ring of ligand interacts strongly via π-stacking interaction with Trp86. Tri-fluoro-phenyl of ligand is docked deeply into the cavity formed by Phe112, Ile198, Trp248, Tyr251, Asn252 and Leu255 residues.

Maraviroc (IC_50_ = 0.56 nM) was first identified as potent CCR5 antagonists by Pfizer pharmaceutical [48]. FDA has licensed this compound as a potent and orally bioavailable compound and has been commercially available since August 2007 for HIV-1/AIDS chemotherapy. In this study, Maraviroc had established crucial interactions with the binding pocket of CCR5. Salt bridge contact was observed between tertiary ‘N’ of the ligand and Glu283 of CCR5 at a distance of 4.55 Å. Tyr37 makes a hydrogen bond with triazole ‘N’ of Maraviroc (1.88 Å), and another hydrogen bond interaction was observed with carboxamide ‘O’ of Maraviroc against Tyr108 (2.17 Å). The isopropyl group of the triazine ring is situated deep inside the pocket formed by the hydrophobic residues Trp86, Tyr108, and Met287. The phenyl ring of the ligand docks into a cavity formed by residues Tyr108, Phe109, and Ile198. The para-difluoro-cyclohexane ring of ligand docks into a hydrophobic pocket determined by Phe112, Phe113, Ile198, Tyr251, and Leu255. The base of the pocket is formed by the highly hydrophobic residues (Phe112 and Phe113). A central fused bi-cyclic ring interacts hydrophobically with the Trp86, Tyr108, and Thr167 residues. The docked pose of Maraviroc within the CCR5 pocket is shown in Figure 8a.

#### Docking study with SCH-C

SCH-C (SCH-351125) was identified as a potent CCR5 antagonist by Schering-Plough Research institute in 2001 [49] with CCR5 activity at 0.69 nM. To gain insight into how SCH-C interacts with CCR5, a molecular docking study was performed. SCH-C interacts with CCR5 through hydrogen bond and hydrophobic interactions. Salt bridge contact was observed between the tertiary ‘N’ of SCH-C and CCR5 Glu283 at a distance of 4.67 Å. It seems that the salt bridge contact acts like an anchor to hold the ligand in the receptor cavity. Hydrogen bond interaction was observed between the piperidine-N-oxide of SCH-C and the Tyr37 of CCR5. Moreover, this piperidine-N-oxide seems to interact through the π - π stacking interactions with Trp86 of CCR5. Another π - π stacking interaction was observed with terminal 4-Br-phenyl of SCH-C and Phe112. The 4-bromophenyl moiety of ligand docks deeply in the receptor pocket lined by residues Phe112, Ile198, Trp248, Tyr251, Asn252, and Leu255. SCH-C interacts through hydrophobic interactions with the receptor pocket lined by residues Tyr37, Trp86, Tyr89, Tyr108, Phe109, Phe112, Gly163, Gln194, Ile198, Trp248, Tyr251, Asn252, Leu255, and Glu283. The docked pose of SCH-C with the CCR5 binding site is shown in Figure 8b.

#### Docking study with TAK779

TAK779 was discovered by Takeda pharmaceuticals as a potent anti HIV-1 agent targeting CCR5. Biological assays revealed CCR5's antagonism potency against CHO (1.4 nM) [50]. TAK779 interacts through hydrophobic interactions. Strong salt bridge contact between quaternary ‘N’ of TAK779 and Glu283 of CCR5 is observed at a distance of 3.95 Å. Hydrogen bond interaction was formed between the central amide carbonyl of TAK779 and Thr167. The docked pose revealed that TAK779 is oriented as a L-shape inside the binding pocket. A T-shaped interaction between the Tyr251 and the fused ring of TAK779 is present. A 4-methylphenyl substituent on the fused ring docks into a cavity formed by Phe112, Phe113, Ile198, Tyr251, Asn252, and Leu255. The base of this pocket is formed by the highly hydrophobic residues such as Phe112 and Phe113. TAK779 docks in the binding pocket lined by residues Tyr37, Trp86, Tyr89, Phe112, Phe113, Gly163, Thr167, Ile198, Gln194, Tyr251, Asn252, Leu255, Gln280, and Glu283. The docked pose for TAK779 along with residues is shown in Figure 8c.

#### Docking study of Vicriviroc

Discovery and characterization of Vicriviroc as potent CCR5 antagonists was done by Strizki et al. [51]. These authors reported Vicriviroc to be 2–40 folds more potent that SCH-C. Presently, a docking study was performed to discern the molecular mechanism of interaction between the ligand and the protein. The docked pose revealed that the ligand-protein interaction is mainly hydrophobic. The tertiary ‘N’ of Vicriviroc makes salt bridge contact with Glu283 at a distance of 4.87 Å. The hydrophobic 4-Trifluoro-phenyl part of Vicriviroc docks deeply into a cavity formed by residues Phe112, Ile198, Trp248, Tyr251, Asn252, and Leu255. Another observed hydrophobic interaction is between the pyrimidine ring of the ligand and Trp86, mainly a π - π stacking interaction. Vicriviroc docks into a receptor pocket lined by amino acid residues Tyr37, Trp86, Tyr89, Tyr108, Phe109, Phe112, Ala159, Thr167, Ile198, Gln194, Trp248, Tyr251, Asn252, Leu255, and Glu283 (Figure 8d).

### Comparative analysis of CCR2 and CCR5

We compared the sequences of CCR2 and CCR5, and found 66% sequence identity. They also share 82% identity in their active sites. From the alignment (materials **S7**) we found that most of the residues are conserved. Since dual targeting of CCR2 and CCR5 is of prime importance in current drug discovery, we moved our focus towards the binding site of these receptors. We superimposed the binding sites of both receptors and analyzed the variation of residual information. Our analysis revealed that almost all the residues are identical except three residues. The varying residues in CCR2/CCR5 are Ser101/Tyr89, His121/Phe109, and Arg206/Ile198; these makes difference in their electrostatic properties. More specifically, Ser101 is hydrophilic and Tyr89 is hydrophobic in nature. Similarly, His121 and Arg206 are hydrophilic, whereas Phe109 and Ile198 are hydrophobic in nature. While designing dual inhibitors one may consider this variation of active sites residues for potent inhibition of dual targets. Mutational studies on these residues could also be effective. The superimposed binding site of CCR2/CCR5 is shown in materials **S8**.

### Docking study of dual antagonists into the binding site of CCR2 and CCR5

**Figure 9:**
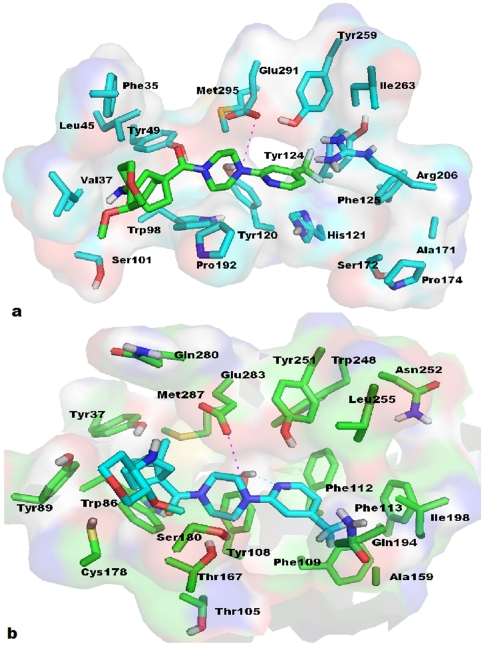
Docked models of dual inhibitor (compound 19). (a) Docking model of compound 19 in CCR2 is shown in transparent surface. Salt bridge interaction of tertiary nitrogen with Glu291 is shown by dotted magenta lines, whereas hydrogen bond interaction of ligand-fluorine with Arg206 is shown by dotted cyan line. Side chains of interacting residues of CCR2 are shown in cap stick (cyan color), while ligand is shown in cap-stick with green color for carbon. (b) Binding mode of compound 19 in CCR5 cavity. Salt bridge interaction of tertiary nitrogen with Glu283 is shown by dotted magenta lines, whereas hydrogen bond interaction of pyridine nitrogen with Tyr108 is shown by dotted cyan line. Side chains of interacting residues of CCR5 are shown in cap stick (green color), while ligand is shown in cap-stick with cyan color for carbon. Trifluoromethyl group of ligand was docked into the hydrophobic cavity.

A series of antagonists targeting both CCR2 and CCR5 has been proposed [21]. Among the series of potent inhibitors, compound 19 was shown to be more potent and inhibited both CCR2 (3 nM) and CCR5 (5.3 nM). To gain crucial information about interaction between this compound and the receptor (CCR2 and CCR5), compound 19 was docked into the binding site of these receptors. The binding mode of this compound inside the receptor active sites was analyzed. The tertiary nitrogen of the ligand forms a salt bridge (i.e. electrostatic interaction) contact with the crucial acidic residue (CCR2-Glu291; CCR5-Glu283). The distance between the tertiary nitrogen of the ligand and the acidic residue is 3.97 Å for CCR2 and 4.48 Å for CCR5. These interactions are likely necessary for high affinity binding. Structural activity relationship study of derivatives of this compound showed that replacement of 4-[3-(trifluoromethyl)phenyl]piperidine by 1-[3-(trifluoromethyl)phenyl]piperazine enhances inhibitory activity 52-fold against CCR2 [21]. The tertiary ‘N’ present in the piperazine ring markedly influences activity, explaining the importance of the tertiary ‘N’ in the present study. Our study implies that interaction occurs through the salt bridge contact with acidic residues. We also found that the trifluoromethyl substitution close to the tertiary nitrogen make them able to interact with Arg206/Ile198 of CCR2/CCR5. The binding mode of compound 19 inside the binding site of CCR2 and CCR5 is shown in Figure 9a and 9b, respectively. The binding poses of this dual antagonist indicate that filling the hydrophobic cavities of both CCR2 and CCR5 would be necessary to develop more potent dual antagonists. Binding energy of dual antagonist is given in materials **S6**.

### Binding patterns of docked molecules against CCR2 and CCR5 models

Our binding pattern of drug matches partially to that of CXCR4 ligand (IT1t), and it overlaps with other GPCR ligands such as, retinal (rhodopsin), carazolol (β_2_AR) and ZM241385 (A_2A_AR) (materials **S9**). Materials **S9a** was generated with the alignment of 1U19 (rhodopsin), 2RH1 (β_2_AR), 3EML (A_2A_AR), Teijin with CCR2 model and TAK779 with CCR5 model over the template structure (CXCR4). Materials **S9b** was generated as a hypothetical model of materials S9a. In our docked model, Teijin (white carbon, materials **S9a**) was partially overlapped with the native ligands IT1t (3ODU), and ZM241385 (3EML), and it also bound in TM1, TM2, TM3, TM4 and TM7 as IT1t do. Also, Teijin overlaps the binding site of CVX15 peptide (native peptide ligand of 3OE0) at the TM3, TM4 and TM5 with close contacts of Tyr120, His121, Pro174 and Arg206. It was also observed an essential salt bridge interaction with the Glu291 of CCR2.

TAK779 [green carbon, materials **S9a**] bound to CCR5 model in a L-shaped orientation which cover the binding sites of GPCRs ligands such as retinal, carazolol, ZM241385, IT1t and CVX15. Quaternary ammonium nitrogen of TAK779 interacts with the Glu283 of CCR5 through salt bridge contact. However, TAK779 binds in a pocket formed by residues of TM1, TM2, TM3, TM5, TM6 and TM7. Docked pose of TAK779 indicates that, it penetrates deep into the pocket formed by TM5, TM6 and TM7 where retinal binds with bovine rhodopsin (1U19). Furthermore, the docked models of other potent antagonists used in this study expressed the similar binding pattern of above mentioned ligand molecules (Teijin and TAK779). Our docked poses overlap the binding sites of these co-crystal ligands and may hinder the activation of receptors. Multiple ligand sites in GPCR shows that the plasticity of binding site. Our docking models of different inhibitors for CCR2 and CCR5 explain the phenomenon of binding site plasticity.

## Discussion

CCR2 and CCR5 are two CC chemokine receptors that are important players in the trafficking of monocytes/macrophages and in the functions of other cell types relevant to disease pathogenesis [19], [20]. Clinical studies suggested that targeting just a single receptor may not be adequate enough for efficacy. Considering the importance of CCR2 and CCR5, the need for developing dual target antagonists is of prime concern. Because of the lack of structural information, targeting both CCR2 and CCR5 receptors has been difficult. In the absence of structural information, ligand-based approaches have proven to be especially useful for G protein-coupled receptor (GPCR) [52]. However, in the absence of X-ray structure, homology modeling could be an important alternative and we implemented a combined ligand and structure based analysis in this study.

Only a few studies have been reported concerning modeling of the structure of CCR2 through a comparative modeling approach [40], [53]–[55]. All the reported homology models were developed using the traditionally used bovine rhodopsin and the recently reported β_2_-adrenergic receptors as templates. However, in the case of CCR5, most of the reported models involved the use of the traditional bovine rhodopsin structure [56]–[61]. We also studied CCR2 and CCR5 using in silico methodologies [62], [63] and both these structures will be important for modelers as well as experimentalists in the scientific community. With the availability of the recently reported close homolog, CXCR4 [23], modeling of CCR2 and CCR5 was performed.

One of the main advantages of CXCR4 structure over bovine rhodopsin is that it has higher sequence identity as well as a larger binding pocket. Kimura et al. explained the importance of expansion of binding site for the models developed based on bovine rhodopsin as the template [54]. From Kimura's report it is obvious that the binding site is small in case of bovine rhodopsin. So, we estimated the binding pocket volume of recently reported CXCR4 (1137 Å^3^) and β_2_-adrenergic (1145 Å^3^) receptor structures. It implies that the binding pockets are comparatively big. Secondly, this template (CXCR4) is more closer with the sequence identity of 35.4% with CCR2 and CCR5, which is quite higher than bovine rhodopsin (23.3%), human β_2_AR (25.4%) and human A_2A_AR (22.2%) receptor templates. Hence this higher identity implies that the CXCR4 template suits better for modeling study.

The molecular docking study was carried out using the modeled receptor structure. Some of the potent antagonists of CCR2 and CCR5 were docked into the proposed binding sites. The binding site of CCR2 [4], [40] and CCR5 [43]–[48] were developed according to previously proposed sites and were in good agreement with already published results. We have identified residues in the previous reports. In addition, we found some important residues that are likely to be crucial in antagonism (CCR2 and CCR5).

Specifically, the obtained docking results for CCR2 antagonists are well correlated with the previous site directed mutagenesis study. It also shows that hydrogen bond interaction is more important for CCR2 antagonists and in accordance with previous reports [40], [64]. Glu291 has been established as a crucial residue for the activity of CCR2 antagonists through mutational studies, and our results confirmed the importance of Glu291 in the active site which formed salt bridge contacts with the antagonists [4], [40]. Besides, our study implies that Tyr120 and His121 may also be crucial for CCR2 antagonists, because Tyr120 and His121 form hydrogen bonds with the ligand molecules. This result complements previous results [40], [64]. It was also observed that Y49, Trp98, Tyr120 and Glu291 forms tight aromatic cluster to accommodate Teijin into CCR2 cavity [65] which is in line with our results. In addition, the docking studies indicated that Ser101, Ala102, Arg206, and Thr287 might also be crucial through our docking studies and mutational studies on these residues might be effective.

Similarly, docking result of CCR5 antagonists are well correlated with that of the previous site directed mutagenesis studies. Our docking study revealed that Glu283 is an important residue in CCR5 antagonism, bolstering prior mutational studies [43]–[47]. Docked poses of all the four inhibitors (MVC, SCH-C, TAK-779, and Vicriviroc) indicated that ligands were bound tightly in the active site. Additionally, we found some important residues in active site, which might be crucial for CCR5 antagonism. Close interactions for all four antagonists (MVC, SCH-C, TAK-779 and Vicriviroc) were observed with Tyr89 (TM2), Gly163 (TM4), Thr167 (TM4), Asn252 (TM6), and Leu255 (TM6). Tyr37, Trp86, Tyr108, Ile198, Glu283 are important in CCR5 antagonism [43]–[47], [61], [66]. Interactions were mainly hydrophilic and hydrophobic in origin. Further mutational studies on these residues might be effective to locate the contribution of these residues towards CCR5 antagonism. Recent report on TAK779 modeling by Peterlini et al., [67] showed that positively charged quaternary ammonium nitrogen of TAK779 shows ionic interaction with the Glu283, which is in line with our docked model.

Though our defined binding sites for both receptors are similar to the previous results, there may be possibility of some limitations with the current strategy of pose selection as we selected our docked modes based on the top cluster, previous mutagenesis results and binding energy of inhibitors. However, our docked models identified the corresponding residues in TM1, TM2, TM3, and TM7 with additional residues from the TM5 and TM6. Our binding modes of the representative compounds Teijin and TAK779 (CCR2 and CCR5) are in agreement with the previously reported results in the literature [64]. Location and interactions of identified residues are in line with the reported results in literature, thus corroborates the reliability of our results.

We also compared the binding site residues of CCR2 and CCR5. Most of the residues are similar. However, varying residues in active sites of CCR2/CCR5 such as Ser101/Tyr89, His121/Phe109, and Arg206/Ile198 were observed. Site-directed mutagenesis studies on these residues can also be effective. We docked a highly potent dual antagonist into the active site of CCR2 and CCR5. The docked poses revealed that tertiary ‘N’ of the piperazine ring makes salt bridge contact with the acidic residues of CCR2/CCR5 and is important for antagonism. We furthermore found that trifluoromethyl substitution of ligand, which is hydrophobic as well as electronegative in nature and which is crucial for inhibiting both CCR2/CCR5. Docked poses revealed that this group interacts with Arg206 of CCR2 and Ile198 of CCR5. Introducing a bulky group to this dual antagonist would increase binding affinities while substituting an electronegative group would differentiate CCR2 and CCR5.

## Supporting Information

Materials S1 **Ramachandran plot of the CCR2 model obtained before MDS.** The different color coding indicates most favored (red), generously allowed (dark yellow), additionally allowed (light yellow), and disallowed (white) regions.
(TIF)

Materials S2 **Ramachandran plot of the CCR5 model obtained before MDS.** The different color coding indicates most favored (red), generously allowed (dark yellow), additionally allowed (light yellow), and disallowed (white) regions.
(TIF)

Materials S3 **ProSA energy plot for the CCR2 and CCR5 models before and after MD simulation.**
(TIF)

Materials S4 **Homology model of CCR2 obtained after refinement by MDS.** The TM domain regions are colored in red and the loop regions are colored in green.
(TIF)

Materials S5 **Homology model of CCR5 obtained after refinement by MDS.** The TM domain regions are colored in red and the loop regions are colored in green.
(TIF)

Materials S6 **Binding energy of all the docked inhibitors by Autodock.**
(XLSX)

Materials S7 **Alignment obtained between the CCR2 and CCR5 sequences for sequence analysis.** Identical residues are marked as (*), similar regions are marked as (:).
(TIF)

Materials S8 **Superposition of varying residues in the active sites of CCR2 (cyan) and CCR5 (magenta).** All the TM's are labeled by blue color on the top of helices.
(TIF)

Materials S9 **Superposition of the GPCRs ligand.** (a) Binding sites of the GPCRs were mapped computationally. X-ray structures of bovine rhodopsin (1U19), β_2_AR (2RH1), A_2A_AR (3EML) were aligned over recent CXCR4 (3ODU) structure. As well as the CCR2 and CCR5 model with docked Teijin and TAK779 are aligned over 3ODU. Aligned ligands were shown; retinal in yellow carbon, carazolol in brown carbon, ZM241385 in magenta carbon, IT1t in cyan carbon, teijin in white carbon and TAK779 in green carbon. (b) Hypothetical model of overlapping binding sites were generated.
(TIF)
