# Supplementary figures and images for: Structural Insights from Binding Poses of CCR2 and CCR5 with Clinically Important Antagonists: A Combined In Silico Study
Source: PLoS One. 2012 Mar 27;7(3):e32864. doi: 10.1371/journal.pone.0032864 (PMC3314010; doi:10.1371/journal.pone.0032864)

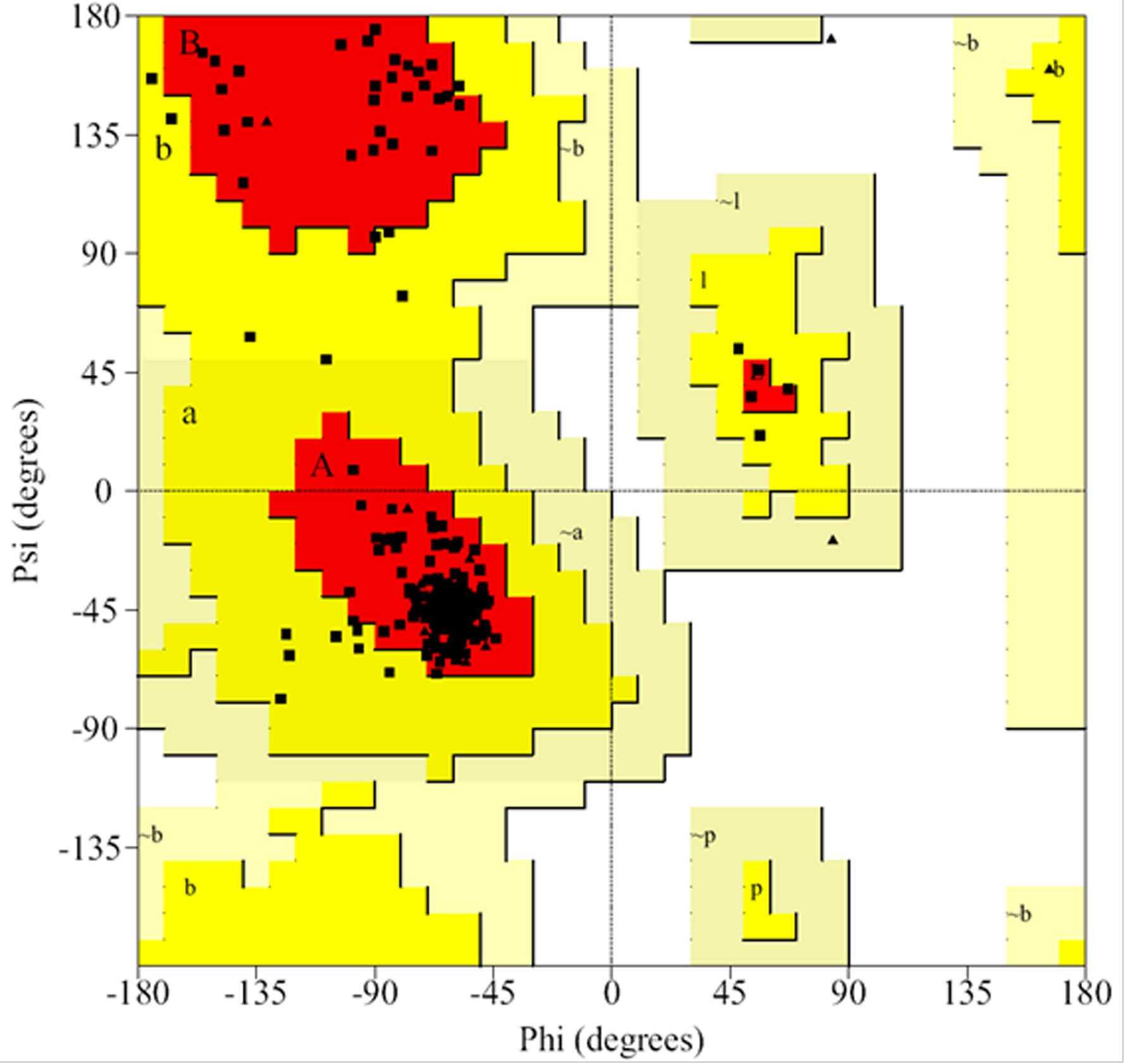

Supplement: Materials S1 — Ramachandran plot of the CCR2 model obtained before MDS. The different color coding indicates most favored (red), generously allowed (dark yellow), additionally allowed (light yellow), and disallowed (white) regions. (TIF) [file pone.0032864.s001.tif]

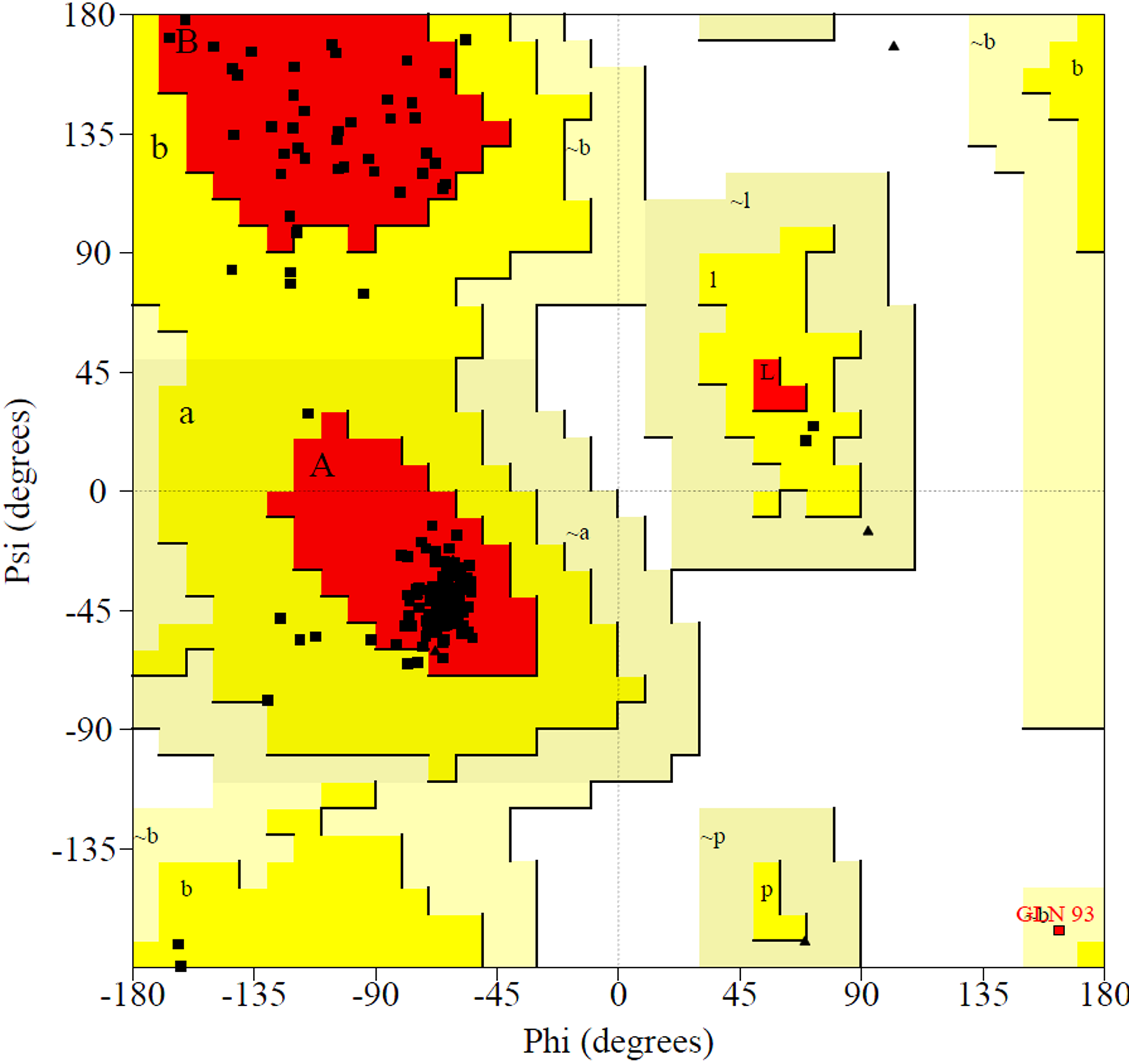

Supplement: Materials S2 — Ramachandran plot of the CCR5 model obtained before MDS. The different color coding indicates most favored (red), generously allowed (dark yellow), additionally allowed (light yellow), and disallowed (white) regions. (TIF) [file pone.0032864.s002.tif]

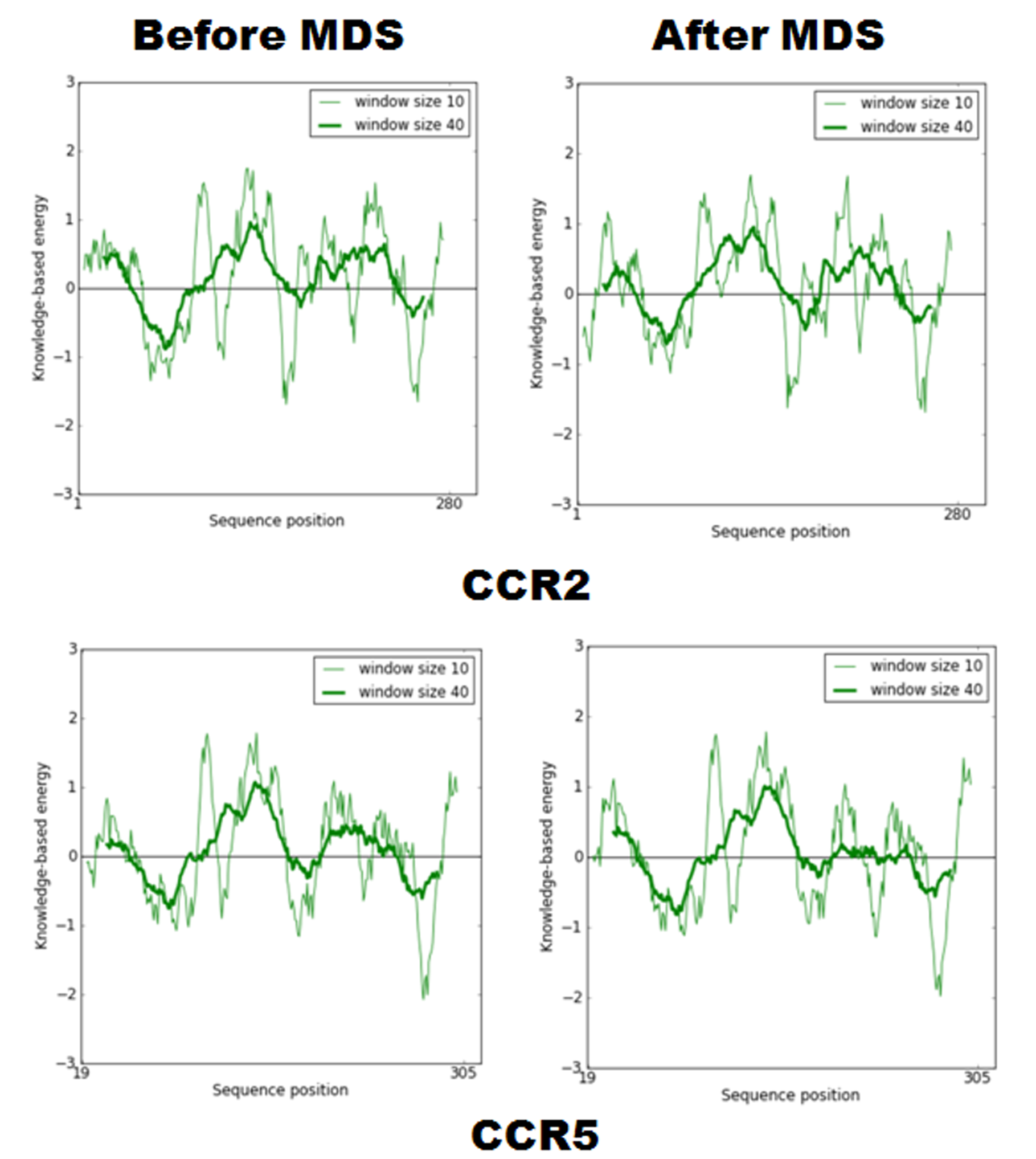

Supplement: Materials S3 — ProSA energy plot for the CCR2 and CCR5 models before and after MD simulation. (TIF) [file pone.0032864.s003.tif]

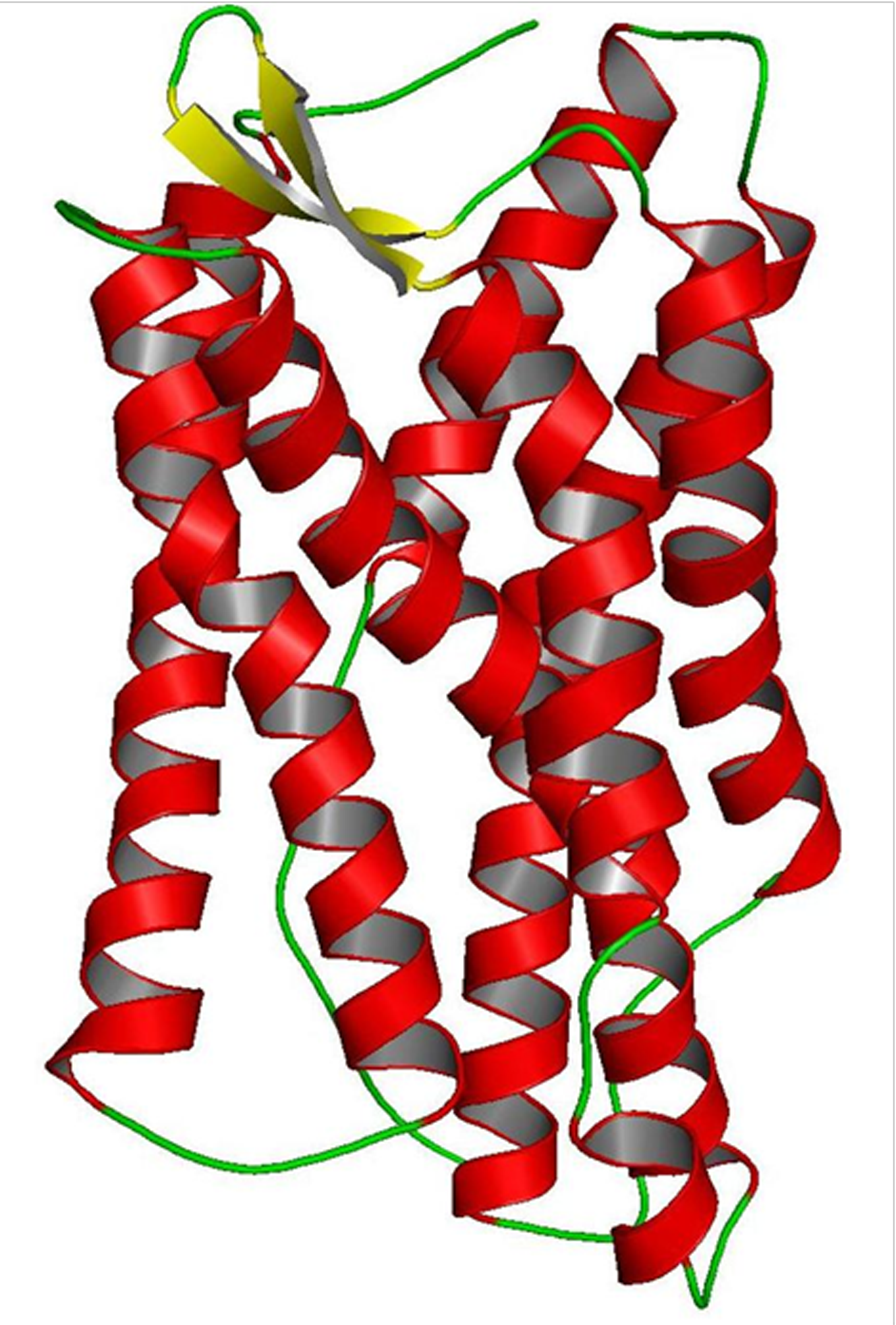

Supplement: Materials S4 — Homology model of CCR2 obtained after refinement by MDS. The TM domain regions are colored in red and the loop regions are colored in green. (TIF) [file pone.0032864.s004.tif]

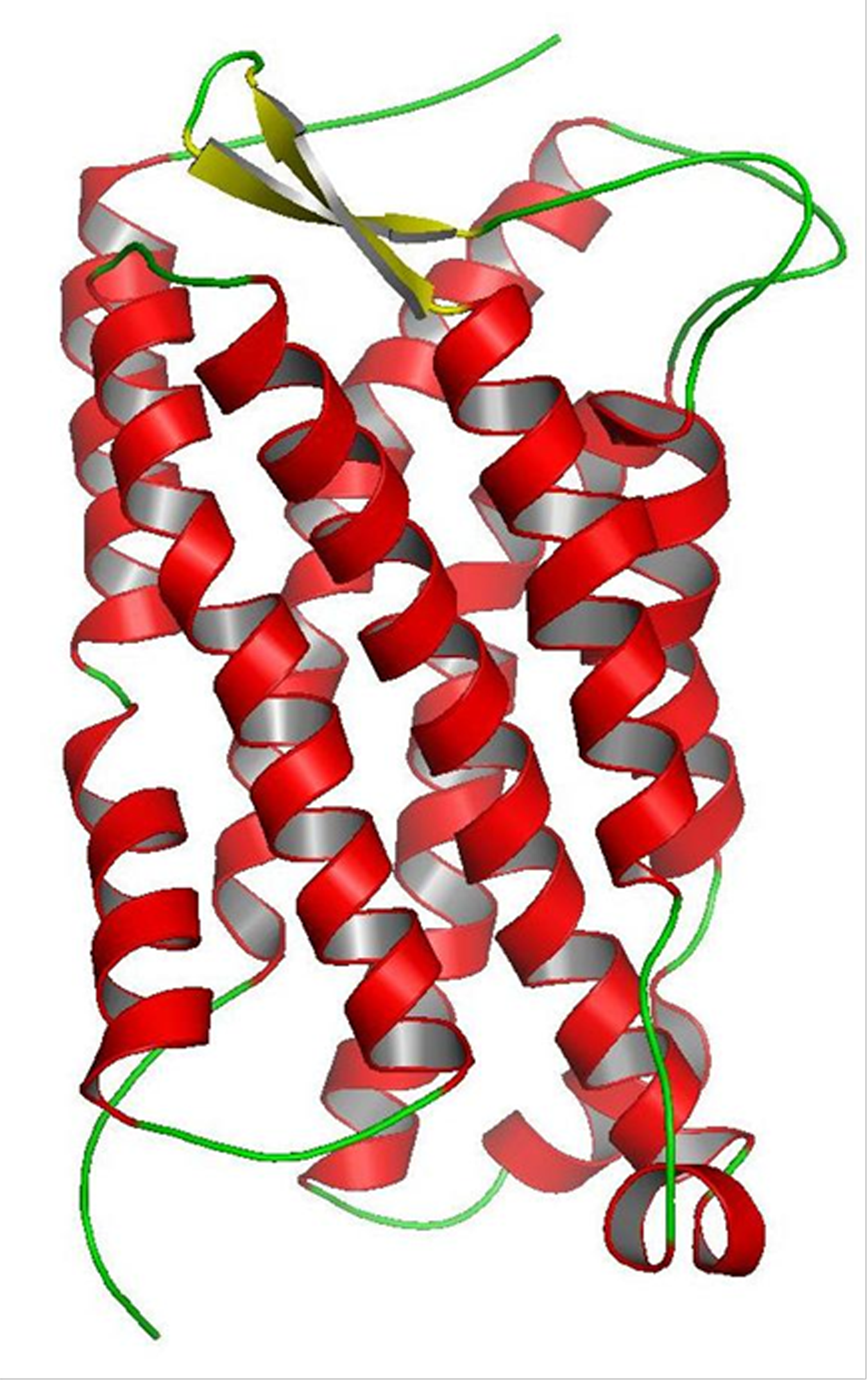

Supplement: Materials S5 — Homology model of CCR5 obtained after refinement by MDS. The TM domain regions are colored in red and the loop regions are colored in green. (TIF) [file pone.0032864.s005.tif]

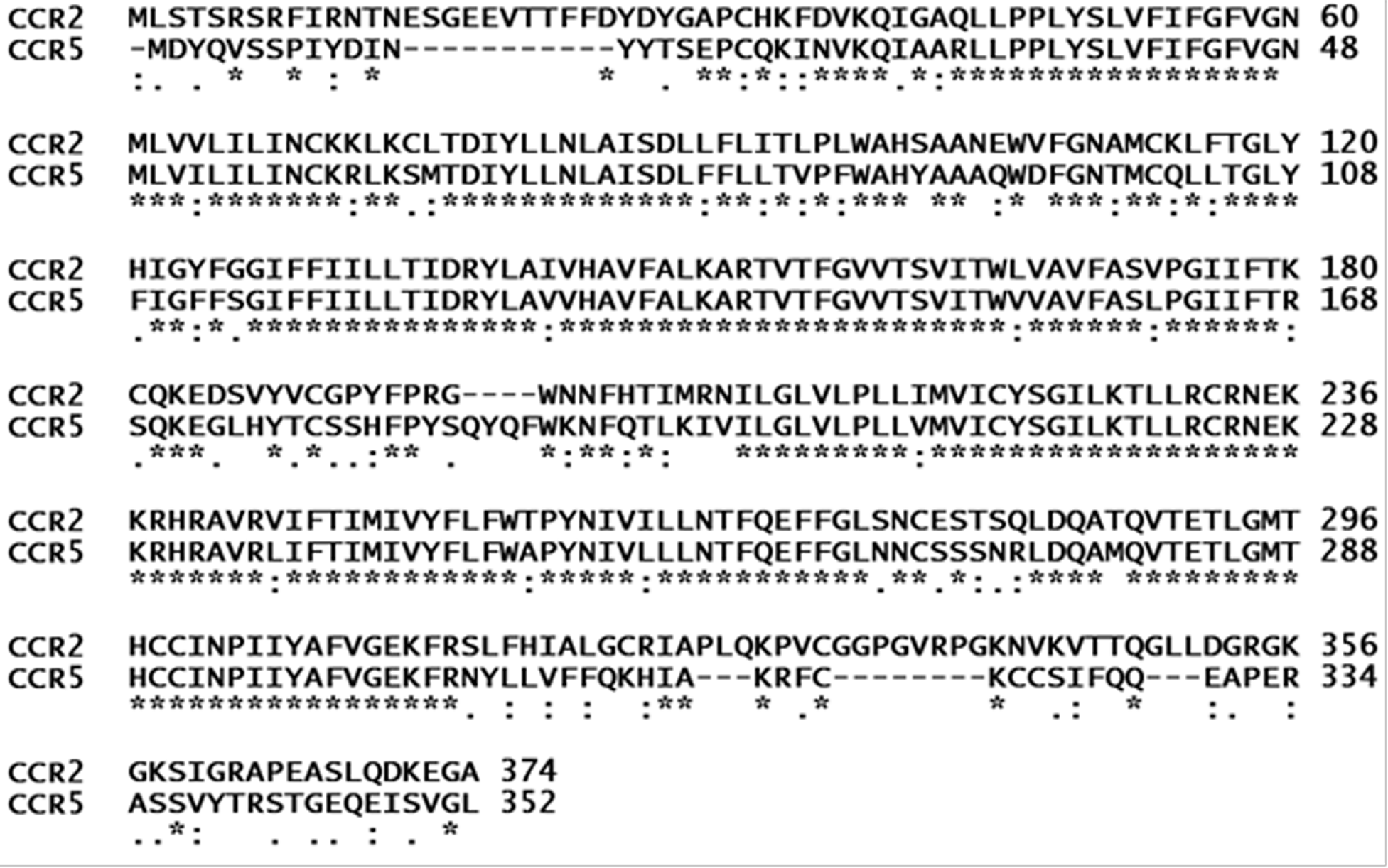

Supplement: Materials S7 — Alignment obtained between the CCR2 and CCR5 sequences for sequence analysis. Identical residues are marked as (*), similar regions are marked as (:). (TIF) [file pone.0032864.s007.tif]

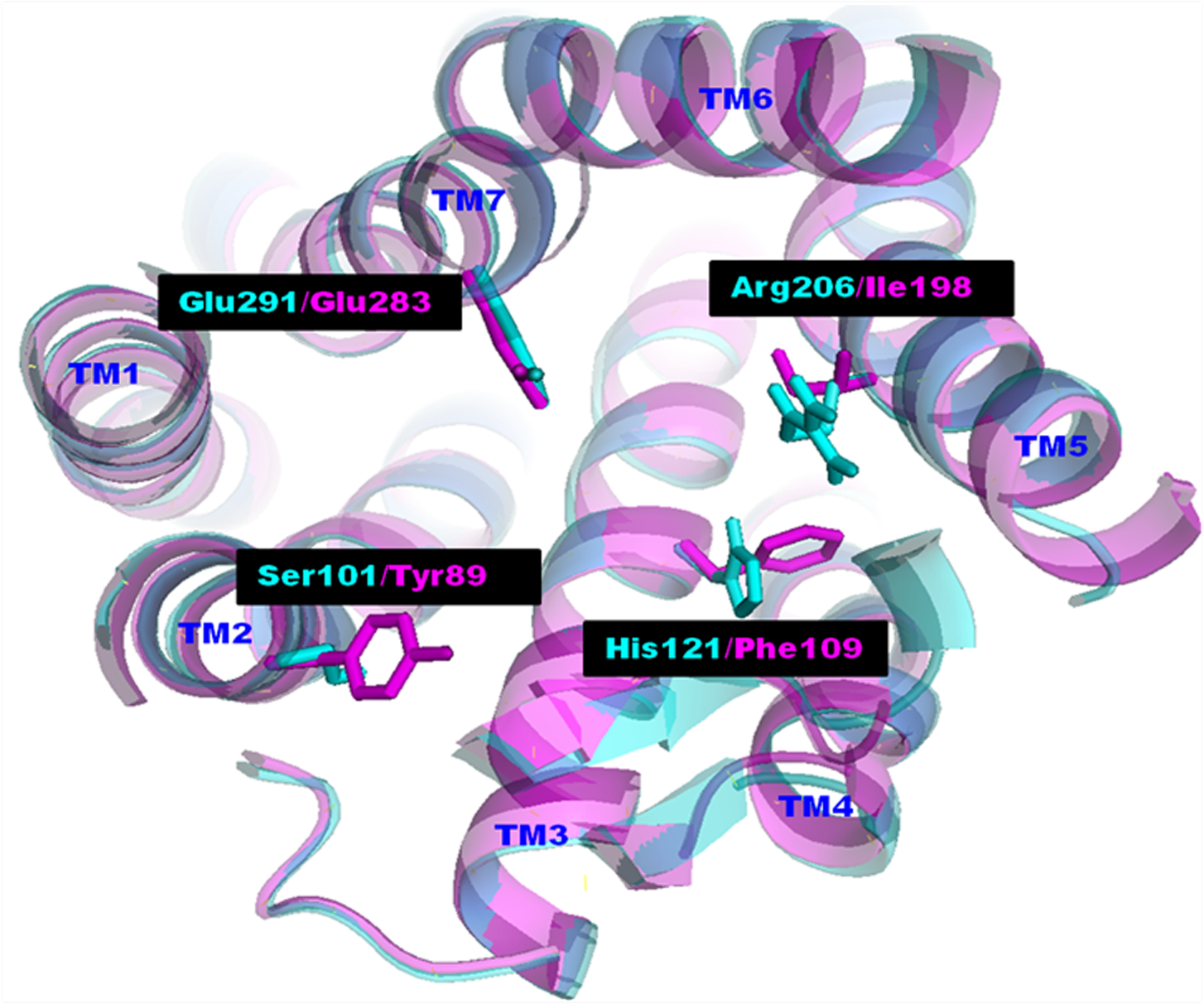

Supplement: Materials S8 — Superposition of varying residues in the active sites of CCR2 (cyan) and CCR5 (magenta). All the TM's are labeled by blue color on the top of helices. (TIF) [file pone.0032864.s008.tif]

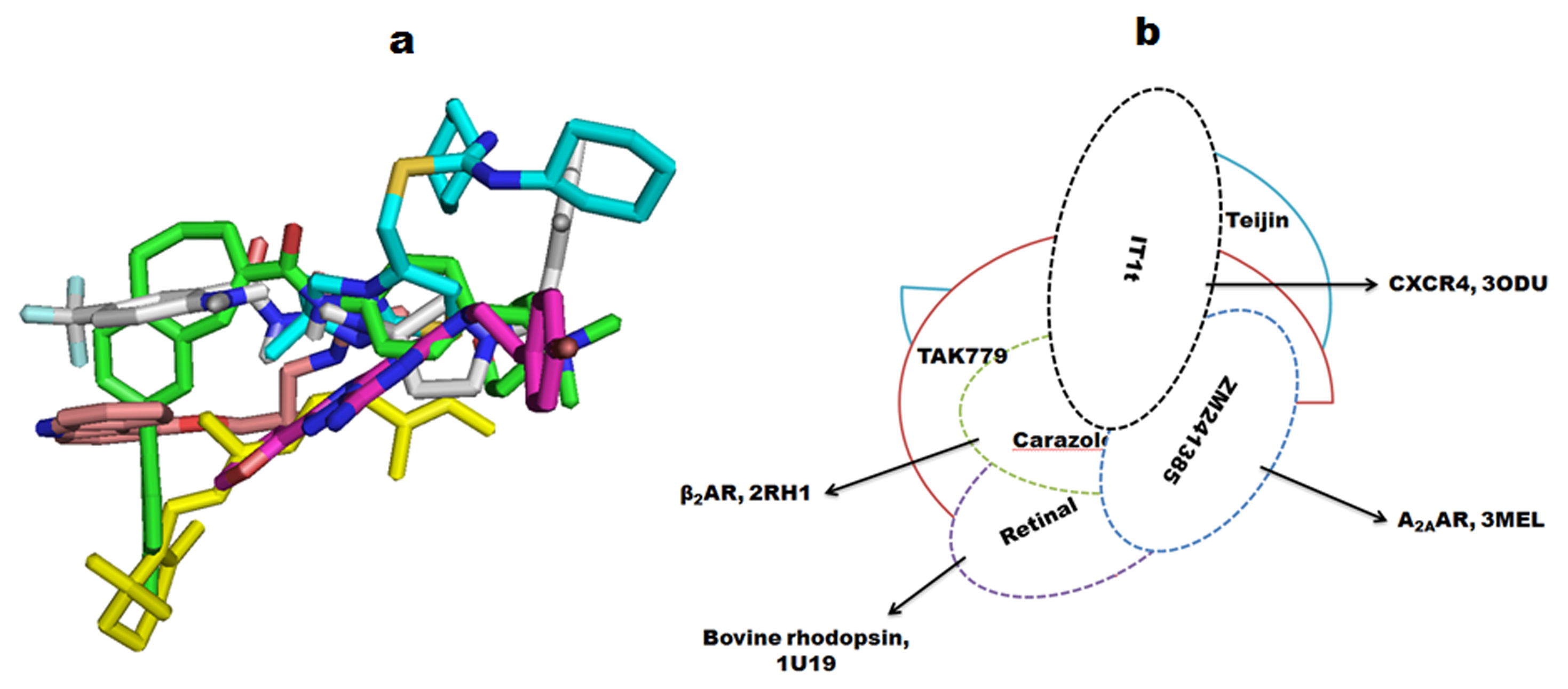

Supplement: Materials S9 — Superposition of the GPCRs ligand. (a) Binding sites of the GPCRs were mapped computationally. X-ray structures of bovine rhodopsin (1U19), β2AR (2RH1), A2AAR (3EML) were aligned over recent CXCR4 (3ODU) structure. As well as the CCR2 and CCR5 model with docked Teijin and TAK779 are aligned over 3ODU. Aligned ligands were shown; retinal in yellow carbon, carazolol in brown carbon, ZM241385 in magenta carbon, IT1t in cyan carbon, teijin in white carbon and TAK779 in green carbon. (b) Hypothetical model of overlapping binding sites were generated. (TIF) [file pone.0032864.s009.tif]
